# Evolution of the Concept of Sepsis Scoring Systems in Pediatrics to Predict Mortality and Outcomes

**DOI:** 10.7759/cureus.74725

**Published:** 2024-11-29

**Authors:** Reshmi Mishra, Amit R Rup, Bharti Aggarwal, Jyoti Ranjan Behera

**Affiliations:** 1 Pediatrics, Kalinga Institute of Medical Sciences, Bhubaneswar, IND

**Keywords:** critically ill patient, picu, prism, psofa, sepsis scoring

## Abstract

Sepsis continues to be a major contributor to illness and death in children, necessitating effective risk assessment tools. Incidence of pediatric sepsis in intensive care units is on increasing trend. Over the years, the concept of sepsis scoring systems has evolved to enhance the prediction of outcomes and mortality in pediatric age group. To better identify sepsis and septic shock status in the pediatric age group, various sepsis scores were developed. Properly applying these scores can significantly enhance timely decision making and ultimately reduce mortality rates. Selecting appropriate score should match the settings where they were designed. Scoring system is broadly categorized into two types - prognostic scores and descriptive or outcome scores. To improve care for critically ill children, it is important to develop tools that can better predict long-term mortality and morbidity and identify factors related to intensive care related events. This review article aims to discuss the evolution of various sepsis scoring systems, highlighting their development over time and their practical utility in clinical settings.

## Introduction and background

Sepsis is an acute emergency condition and is one of the major causes of mortality in pediatric age group globally. Mortality due to sepsis is around 5.3 million per year globally [1]. The body's inflammatory response becomes dysregulated in cases of infections caused by bacteria or virus, leading to sepsis. If left untreated, it can progress to tissue destruction, major organ dysfunction, and ultimately, death. The incidence of death due to sepsis in pediatric intensive care units (PICU) is on an increasing trend [2]. To reduce mortality, timely recognition of sepsis is important. Identifying infection early in children having systemic inflammatory response syndrome (SIRS) could serve as a useful indicator for guiding treatment. Identifying sepsis and septic shock in the pediatric age group more effectively requires the use of accurate and definitive criteria to assess its progression. Therefore, many scoring systems were developed over time. The assessment of quality of care is often done using scoring systems. This helps in evaluating and making changes to complex care systems, enhancing patient outcomes and predicting morbidity and mortality. There are different types of scoring systems, which can be categorized into two main groups: prognostic scores, which estimate the risk of mortality at admission to the PICU, and descriptive scores or outcome scores, which provide information about the progression of illness after hospitalization [3]. Physiologic stability index (PSI), pediatric risk of mortality (PRISM), and pediatric index of mortality (PIM) are the prognostic scores. Pediatric logistic organ dysfunction (PELOD) score, pediatric multiple organ dysfunction scores (P-MODS), and pediatric sequential organ failure assessment (pSOFA) are examples of descriptive scores (Table 1). This review aims to describe various sepsis scoring systems used in PICU and provide their usefulness and limitations.

**Table 1:** Categorization of sepsis scoring systems PSI: physiologic stability index; PRISM: pediatric risk of mortality; PIM: pediatric index of mortality; PELOD: pediatric logistic organ dysfunction score; PMODS: pediatric multiple organ dysfunction score; pSOFA: pediatric sequential organ failure assessment

Prognostic scores	Descriptive or outcome scores
PSI	PELOD
PRISM	PMODS
PIM	pSOFA

## Review

Physiologic stability index

Using adult-derived scoring systems for assessing physiological changes in infants and children in pediatric intensive care can indeed be challenging. Infants and children have unique responses to illness and treatment, and their normal physiological parameters differ considerably from those of adults. To address this, a group of pediatric intensivists in 1984 validated the pediatric severity index (PSI) score by comparing it with two other methods, the therapeutic intervention scoring system (TISS) and the clinical classification system (CCS) [4-6]. It reflects the severity of acute illness in infants and children getting admitted to the PICU [4]. The severity of illness is evaluated by PSI by quantifying the degree of derangement in 34 variables across seven major physiological systems. Scoring was based on the highest abnormal value of a variable observed within a 24-hour period. A score of 1 was assigned to each variable, indicating an abnormality worth concern but not requiring a change in therapy, a score of 3 was given to signify the need for a change in therapy, while a score of 5 indicated a life-threatening situation. Each variable's individual scores were summed to calculate the final PSI score for each. The limitation of this score is it uses lots of variables with subjective scores [4].

Pediatric risk of mortality

PRISM scores are usually used in neonatal to adolescent age groups [7]. Pollack et al. developed the PRISM II score in 1988, which was an updated version of PSI [7]. In the first 24 hours in the PICU, it utilizes 14 parameters (physiological and laboratory data) instead of 34, with a decrease in the number of ranges from 75 to 23, in comparison to PSI. Calculating it was simpler and it provided a more accurate representation of the disease severity compared to PSI. Some studies revealed that PRISM could assess indication of mortality rate compared to the past, which showed that PRISM overestimates mortality [8]. The major limitation was underestimating mortality after cardiac surgery [3].

Developed in 1996, the PRISM III is a pediatric physiology-based index designed to assess the risk of mortality in children [9]. This scoring system, validated across 32 PICUs in North America, utilizes 17 routinely measured physiological variables categorized into 26 ranges to gauge disease severity. Data is collected in the initial 12 and 24 hours of hospitalization, focusing on the most abnormal values for each variable. The PRISM III refined its predecessor by removing ranges with minimal impact on mortality risk and incorporating age-adjusted physiological variable ranges for neonates, infants, children, and adolescents. According to Anjali et al., PRISM III is a robust predictor of mortality and disease severity, with higher scores correlating with increased mortality [10]. However, the PRISM scoring system has three notable limitations: it is time-consuming, the validation site had a high death rate within the initial 24 hours, potentially skewing the results toward diagnosing deaths rather than predicting them, and variability in outcomes may arise depending on the standard and level of management provided in different PICUs.

In 1997, Pollack et al. introduced the PRISM III acute physiology score (PRISM III APS), an expanded version of the PRISM III with 59 ranges across 21 physiological variables [11]. Although this score offers heightened sensitivity to minor physiological changes, it is not recommended for routine quality assessments or individual risk calculations due to its high sensitivity.

PRISM IV was published in 2016 by collecting data from seven North American services between 2011 and 2013 [12]. The data collection uses various time intervals to prevent institutional bias arising from varying unit treatment practices. In PRISM IV, laboratory samples were collected from two hours prior until four hours post-PICU admission, and physiologic variables values were calculated in the initial four hours. The variables were gathered and categorized like that of PRISM III scores.

Pediatric index of mortality

The calculation of PRISM involves using the 14 variables having highest abnormal values within a 24-hour period, so gathering extensive data was challenging. To overcome this problem, the first version of PIM score was developed in 1997, which consists of eight variables gathered during the first hour after getting PICU admission [13]. Some variables not in PRISM were added in PIM, such as mechanical ventilation, a specified diagnosis, and plasma base excess. PIM score has advantages like, it has fewer variables, free of cost and easy to use. One of the major drawbacks of PIM is the “lead time bias,” which means treatment is given before admission to the PICU [13].

 In 2003, PIM 2 was developed, which includes 10 variables [14]. Three variables were added, which tell about the primary reason for admission to ICU. These were admissions for recovery from procedure or surgery, following cardiac bypass, and for low‑risk diagnosis [14]. PIM 3 was developed in 2013, which consists of 10 variables [15]. Variables of PIM 3 were similar to PIM 2 but were reorganized. In PIM 3, the low-risk and high-risk diagnoses from PIM 2 were reclassified into low-risk, high-risk, and very high-risk categories [15]. The PIM 3 score has strengths in its validation population, which is large (consisting of 53112 patients). It also offers quantitative assessment within one hour of admission to the PICU, publishes coefficients for each variable, and provides a death probability equation. The PIM 3 score's primary limitation is that it was developed solely using the variables employed in the PIM 2 score [16].

Pediatric logistic organ dysfunction score

PELOD scores (1999, 2003) were created to assess the organ dysfunction severity in children in PICU and to make prognostic assessments [17,18]. It comprises six organ dysfunctions and 12 variables. The measurements of the variables occur multiple times within a 24-hour period, and the highest value is used to calculate the score for that day (dPELOD). It is done for the first five days of the total stay and is used to calculate the PELODs. PELOD is quantitative, but discontinuous, so may create problem during analyzing statistical data [19].

In 2013, PELOD was updated and validated to PELOD 2, which comprises 10 variables, involving five organ dysfunctions (cardiovascular, neurologic, renal, pulmonary, and hematologic) [20]. The PELOD 2 score differs from the previous version by excluding hepatic organ dysfunction and substituting lactatemia and mean arterial pressure for heart rate and systolic blood pressure in the PELOD score. The data was gathered over a span of eight days in the PICU, including days 1, 2, 5, 8, 12, 16, and 18, as well as during discharge from PICU. The measurement of a variable on a day not included in this set may be overlooked, leading to potential underestimation of the score. This was created and validated using data from just two countries (France and Belgium) that are distinct from other regions of the world [20].

Pediatric multiple organ dysfunction score

It was created and validated in 2005 in a single center of United States, which includes five organ dysfunctions excluding neurological [21]. It has not undergone external validation via publication.

Pediatric sequential organ failure assessment

pSOFA was developed in 2017 for the initial evaluation of Sepsis-3 in children who are critically ill [22]. It was adaptation of the original SOFA score for adult patients through two approaches. First, by utilizing validated cutoffs of PELOD 2 scoring system, age dependent renal and cardiovascular variables can be modified. Second, the respiratory sub-score will be broadened to encompass the SpO2:FiO2 ratio, which serves as an alternative indicator of lung injury. The pSOFA score typically assesses six organ systems, namely respiratory, cardiovascular, renal, coagulation, hepatic, and neurological. Each 24 hours, the worst variable was assigned a sub-score for each system (ranging between 0 and 4 points). Daily pSOFA score was calculated by adding six sub-scores in each 24 hours. Minimum to maximum scores ranged from 0 to 24, with higher scores indicating worse outcomes [22]. The pSOFA scores define pediatric sepsis, assess individual organ dysfunction progression, predict the outcome, and offer a dynamic assessment of disease progression. By using retrospective data, it was done in a single center. Validating pSOFA in a broader, multicenter sample of PICU children was essential [22].

In 2019, a study done by Ghada Mohamed El-Mashad et al. assessed all children in the PICU using SOFA, PRISM, and PIM2 scores, along with SIRS at admission. That study revealed SOFA scores were higher in non-survivors, and mortality increased with high SOFA scores. The cutoff of 3 points on the pSOFA score was found to be more precise in predicting mortality compared to the conventional SIRS criteria and the two-point cutoff suggested by Sepsis-3 [23]. According to Rosida et al, pSOFA is the best in predicting prognosis in case of sepsis in children compared to PRISM, PRISM-III, and PRISM-IV, PIM, PIM 2, PIM 3, PELOD, PELOD 2 and PMODS [2]. One of the studies found pSOFA was better in predicting mortality in children who were critically ill compared to PRISM III [24].

Phoenix sepsis score

Sepsis was defined by the Third International Consensus Conference for Sepsis and Septic Shock (Sepsis-3) as a life-threatening organ dysfunction caused by a dysregulated host response to infection, as indicated by an acute change in total SOFA score of at least two points [25]. Society of Critical Care Medicine (SCCM) developed and validated Phoenix sepsis criteria for sepsis and septic shock in pediatric age group by utilizing a modified Delphi consensus approach, a comprehensive review and meta-analysis, and large international database and survey [26]. The SCCM task force has indicated that a Phoenix sepsis score of two or more points can be used to detect sepsis in suspected patients, indicating potentially fatal impairment of respiratory, coagulation, cardiovascular, and/or nervous systems. Children with septic shock were characterized as children with sepsis who exhibited cardiovascular dysfunction, demonstrated by meeting at least one cardiovascular criterion in the Phoenix sepsis score. These criteria encompassed severe hypotension based on age, a blood lactate level exceeding 5 mmol/L, or the need for vasoactive medication. The Phoenix sepsis criteria for children have demonstrated enhanced performance in diagnosing pediatric sepsis and septic shock when compared to the existing International Pediatric Sepsis Consensus Conference (IPSCC) criteria [27].

Few limitations are there for Phoenix sepsis score [26]. This score does not encompass the full spectrum of biological complexities involved in sepsis, including variations in the host's immune response, the characteristics of pathogens involved, and the contextual factors affecting disease presentation and progression. Since data collected from higher-resource settings were only obtained from children's hospitals of US, it is possible that they can not be applied to other higher-resource nations. As the criteria was developed using a 24-hours presentation window, health care-related sepsis was not excluded. It primarily focuses on death as the main endpoint in children with sepsis but does not talk about morbidity related to infection, and excludes the long-term effects of sepsis on children and their families. Physiological measurements that are dynamic may be able to predict sick children more precisely than static or one-time point evaluations used in the current criteria [26]. Summary of various sepsis scores are depicted in Table 2.

**Table 2:** Summary of various sepsis scoring systems TISS: therapeutic intervention scoring system; PSI: physiologic stability index; PRISM: pediatric risk of mortality; APS: acute physiology score; PIM: pediatric index of mortality; PELOD: pediatric logistic organ dysfunction score; pSOFA: pediatric sequential organ failure assessment

Years	Scores	Components	Advantages	Disadvantages
1974	TISS [5]	Within first 24hrs	One of the initial scores	Age adjusted variables and ranges were absent
1984	PSI [4]	Within first 24 hrs. Physiological system-7. Variables-34. Ranges-75	Presence of age-adjusted variable ranges	Contained large numbers of variables and ranges, so difficult to use
1988	PRISM [7]	Within first 24hrs. Variables-14. Ranges-23	1. Less variable and ranges, so easy to calculate than PSI. 2. Excellent indication of risk of mortality at the end of first 24 hrs. 3.Use as prognostic score in PICU to assess gravity of disease	1. Overestimate the mortality. 2. Age included in explicit variable. 3. Not valid for postoperative cardiac patients
1996	PRISM III [9]	Within first 12hrs, 24hrs. Variables-17.	1. Eliminate some ranges that did not contribute significant mortality risk and add some significant variables. 2. Age included in logically and clinically more convincing	1. Time consuming process. 2. Institutional bias due to different treatment practice between units
1997	PRISM III APS [11]	Variables-21. Ranges-59	More detailed acute physiology score	Not be used routinely for quality assessments or calculating risk of individual patients because it is highly sensitive to small changes in physiological status
2016	PRISM IV [12]	Within first 4hrs	1. No Institutional bias. 2. Sample collected from 2 hrs before until 4 hrs after PICU admission	1. Time consuming compared to PIM score. 2. Complex to calculate
1997	PIM [13]	Within one hr of admission. Variables-8	1. Less variables. 2. Done in 1 hour of admission. 3. Simpler and quicker to use than PRISM, requires minimal data. 4. Early identification of the severity of illness and the timely stratification for interventions	Lead bias - effect of treatment given prior to admission to the PICU
2003	PIM 2 [14]	Variables-10	1. Eliminate lead bias. 2. Can be used for postop cardiac patient and procedure	Constructed only from the variables used in PIM 2 score
2010-11	PIM 3 [15]	Variables-10	Risk factors were reorganized	Estimates initial mortality risk but lacks utility for ongoing assessment of clinical response to treatment
1999	PELOD [17]	Variable-12	1. Reproducible. 2. Excellent discrimination. 3. Comprehensive assessment. 4. Proven predictive value	1. Over-predicts mortality. 2. Has poor calibration and outdated parameters, complexity, less specificity
2013	PELOD 2 [20]	Variables-10	1. Updated parameters. 2. Inclusion of lactate. 3. Simplified scoring. 4. Improved predictive accuracy. 5. Broader validation	1. Learning curve for clinicians. 2. Initial implementation challenges. 3. Less historical data
2017	pSOFA [22]	Variables-6	1. Defining of pediatric sepsis. 2. Assessing individual organ dysfunction progression. 3. Useful for predicting the outcome and offering a dynamic assessment of disease progression	1. While simpler than some other tools, pSOFA still requires multiple organ-specific measures. 2. Relies on lab values and specific measurements, limiting use in resource-constrained settings
2024	Phoenix sepsis score [26,27]	Organ systems-4	Enhanced performance in diagnosing sepsis and septic shock	1. Does not talk about morbidity related to infection. 2. Excludes long-term consequences of sepsis on patients

## Conclusions

PRISM, PIM, and PELOD are effective at predicting short-term outcomes like death in PICUs, but they may not be appropriate for developing countries. Factors such as limited resources, varying patient characteristics, and differences in staff training can impact the accuracy of these scores, where infections and malnutrition are more prevalent. These existing scores are not designed to predict long-term outcomes, which is crucial to know post-PICU discharge status. A new region-specific scoring system should be developed to improve health care in resource-limited settings, and it has to be validated by extensive research before its application to diverse clinical environments.
